# Assessment of aortic stiffness during atrial fibrillation: solutions and considerations

**DOI:** 10.3389/fcvm.2024.1449168

**Published:** 2024-08-29

**Authors:** Kristina Lundwall, Maria Al Nouh, Thomas Kahan, Jonas Spaak

**Affiliations:** Division of Cardiovascular Medicine, Department of Clinical Sciences, Danderyd Hospital, Karolinska Institutet, Stockholm, Sweden

**Keywords:** aortic stiffness, atrial fibrillation, pulse wave velocity, central blood pressure, augmentation index

## Abstract

**Background:**

Methods to assess aortic stiffness are not validated during ongoing atrial fibrillation (AF) We aimed to determine whether aortic stiffness can be assessed reliably in patients during AF.

**Methods and results:**

Carotid-to-femoral and aortic pulse wave velocity (cf/aoPWV), central blood pressure (BP), and augmentation index (AIx) were assessed by a two-site applanation method and a one-site cuff-based oscillometric method in 40 patients with persistent AF and repeated after cardioversion to SR. Mean age was 63 ± 8 years, 73% male, 50% hypertensive. For the two-site method, cfPWV values were slightly higher in AF than in SR (9.3 ± 1.8 vs. 8.5 ± 1.6 m/s, *p* < 0.001), whereas the one-site method provided similar values in AF and SR (10.1 ± 1.5 vs. 10.0 ± 1.8 m/s).The variability indices from the device was higher in AF for the two-site method (SD 2.5 ± 1.7 vs. 1.0 ± 0.5 m/s, *p* < 0.001) but similar in AF and SR with the one-site method (SD 0.7 ± 0.2 vs. 0.6 ± 0.2 m/s). Both methods yielded higher central BP (+4.8/+6.6 and +4.1/+5.7 mm Hg) and lower Aix (−6.8 and −9.1 mm Hg) in AF.

**Conclusions:**

Aortic stiffness can be assessed during AF. Both methods yielded higher central BP and lower AIx in AF, but similar results for PWV in AF and SR, also when adjusted for BP changes. The two-site method showed high variability necessitating repeated measurements. The one-site method showed lower device-calculated variability and needed fewer repeated measurements.

## 1 Introduction

The most common underlying cause of atrial fibrillation (AF) is hypertension (1, 2), and these diseases combined entail a multifold risk of future cardiovascular events (2–4). The prevailing theory is that hypertension accounts for an increased left ventricular load, inducing stiffening and left ventricular hypertrophy, and thereby left atrial dilatation and fibrosis (2, 5). While studies show an association between left ventricular hypertrophy and AF (6), and pathological left atrial function in hypertensive subjects (7), the pathophysiology of the transition to AF is less well studied. Some studies suggest that an increased aortic stiffness may be a more important determinant than peripheral blood pressure (BP) in this process (8, 9). This is supported by studies showing an independent relation between aortic stiffness and future new onset AF (10–12).

Few studies have assessed the importance of aortic stiffness in patients with a diagnosis of AF. One important reason why, is that current methods to assess aortic stiffness have not been validated and are not recommended for patients with ongoing AF (5). Carotid-to-femoral pulse wave velocity (cfPWV) measured by applanation tonometry at two vascular sites is the most validated non-invasive measure of aortic stiffness in terms of prognostic importance (13). This method use ECG triggered time differences between the two vascular sites, and requires a stable heart rhythm for at least 10–20 s (14, 15). Thus, studies in patients with AF using this technique were performed after interventions to restore sinus rhythm (SR) (16, 17). Some studies have also used methodss based on multiple cuffs to estimate systemic arterial stiffness (18). They are, however, not validated for use during arrhythmia, and have demonstrated limited relationship to aortic stiffness (19).

Single cuff-based oscillometric devices use the arterial pressure curve during occlusion to identify the forward- and the reflected arterial pressure peaks, to estimate aortic PWV (aoPWV) in a beat to-beat manner. Such methods to assess aoPWV have been validated to non-invasive and invasive measurements of cfPWV (20, 21). The results using this method relate to the extent of atherosclerotic disease in healthy individuals (22), and preliminary findings show prediction of cardiovascular morbidity and mortality, and all-cause mortality (23, 24).

Non-invasive reliable methods to assess large artery vascular function during AF are needed to further understand the role of aortic stiffness in patients with AF. Beat-to-beat variations with BP measurements during AF show regression towards the mean (25), suggesting that a technique based on several repeated measurements of aortic stiffness indices could be an option during arrhythmia. This study aimed first to study whether aortic stiffness measurements can be performed during persistent AF, and second to assess the reliability and variability of measures of aortic stiffness during AF and after successful electric cardioversion to SR. Thus, intra-individual comparisons in AF and in SR of repeated measurements of cfPWV, central BP and augmentation index (AIx) were performed and indices of variability of measurements were evaluated with a two-site ECG-triggered applanation-based methodology. In addition, we performed repeated beat-to-beat assessments of aoPWV, central BP and AIx with a one-site single cuff-based oscillometric device.

## 2 Methods

### 2.1 Study population

In all, 45 patients with persistent AF scheduled for electric cardioversion at Danderyd University Hospital, Stockholm, Sweden, were invited to participate and accepted participation. Measurements were performed during persistent AF within 1 week before scheduled electric cardioversion, and repeated during SR, between 2 h and 1 week after electric cardioversion. We excluded 5 patients who did not obtain stable SR after the procedure. The study was approved of by the National Ethics Review Authority (# 2020-06355) and was performed in line with Helsinki Declarations of 1974 (as revised in 2013). All patients provided their informed consent.

### 2.2 Procedures

All investigations were performed in the supine position at rest in a quiet room at the Cardiovascular Research Laboratory, Danderyd University Hospital, Stockholm, Sweden. Patients were asked to continue with their prescribed medications but refrain from caffeine and nicotine. Heart rhythm was determined by a standard 12-lead ECG (Cardiolex Medical AB, Solna Sweden) before further examination. Brachial BP was measured with an oscillometric device (Arteriograph, TensioMed Kft, Budapest, Hungary) and an appropriate cuff size as a mean of three recordings. Mean arterial pressure (MAP) was obtained as calculated from the device. Weight and height were measured, and body mass calculated as weight/height^2^. Medical history was reported by the patients and verified against electronic health records.

#### 2.2.1 Assessment of aortic stiffness

Assessments were performed with the two-site ECG-triggered device (SphygmoCor AtCor Pty, West Ryde, NSW, Australia) using applanation tonometry (Millar Instruments, Houston, TX, USA) to determine a pulse waveform from the right radial, carotid and femoral arteries, as described in detail elsewhere (14, 26). The integrated software determined central BP and calculated AIx as the ratio between augmentation pressure (augmentation from reflected wave to late systolic peak) and pulse pressure (PP). The cfPWV was determined by subsequent registrations from the carotid and femoral artery, using the ECG-triggered time difference as pulse wave travel time and the distance calculated by the subtracted distance method (27). We used 10 s recordings from each site, which is within the shorter recommended interval (15), since longer registrations yielded too few approved recordings due to the irregular rhythm. An operator index, given by the software as an indicator of the variability of recorded wave forms, (average pulse height variation, diastolic variation, shape deviation and pulse length variation) of >75% rendered an approved measurement for pulse wave analysis by the device. A standard deviation (SD) given by the software as an indicator of variability in measurements during the registration period of cfPWV <10% was needed for an approved recording. A “not measurable” recording indicated the inability of the device to find a sufficiently stable wave form for calculations.

We also used a one-site beat-to-beat single cuff-based oscillometric device (Arteriograph, TensioMed Kft, Budapest, Hungary). The device uses upper arm suprasystolic vascular occlusion and a piezoelectric sensor to register the forward (left ventricular ejection) and reflected wave form of the central pulse wave, as described in detail elsewhere (21, 26). The torniquet (applied on the right arm) automatically inflates and repeats the assessment of a series of pulse waves/heart beats 3 times. For each inflation, 3 consecutive pulse waves are averaged. From these assessments, the integrated software determined central BP and calculated AIx (as described above). The aoPWV was estimated, by using the return time from forward to reflected pulse wave and the distance from the suprasternal notch to the symphysis (direct measure). Standard deviation (SD) was given by the software, comparing the 3 repeated averaged values of aoPWV. A difference of <1 m/s was needed for an approved recording by the device. A “not measurable” recording (no calculation performed) indicated inability of the device to find a sufficiently stable wave form for calculations.

#### 2.2.2 Number of measurements for each patient

While 2–3 approved measurements are generally recommended for the SphygmoCor and the Arteriograph, this study aimed to evaluate the reliability of measurements in conditions with assumed increased variability due to AF. Thus, we registered the number of all approved, not approved and not measurable recordings for each patient. We aimed for 5 approved measurements with each device on each visit; however, no more than 10 attempts were performed due to the potential discomfort for the patient.

### 2.3 Statistical analysis

Data are presented as mean values ± SD, or as fractions (%), where appropriate. Power calculations for paired comparisons (2 sided α 0.05, β 0.80) to demonstrate a difference of 1 m/s in PWV with a SD of 1.5 m/s required 21 patients. Intraindividual comparisons between measurements in AF and SR were performed by Student´s paired *t*-tests and intraclass correlation coefficients (ICC). To evaluate the potential influence of BP and heart rate (HR) on assessments of stiffness two models were performed. First, the association between stiffness indices (PWV and AIx) and central BP and HR in AF (visit 1) and in SR (visit 2) were investigated by linear regression using stiffness indices as dependent and central BP and HR as independent variables, adjusted for age, body mass index, and concomitant disease (hypertension, diabetes, ischemic heart disease). Second, the impact of changes in central BP (MAP) and HR between visit 1 and visit 2 were investigated, by calculating the difference of these parameters between the visits (value in AF, visit 1, minus the value in SR, visit 2). Linear regressions were then performed with stiffness measurements in AF as independent, difference for central MAP and HR as covariates, and stiffness measurements in SR as dependent variables. SPSS version 27 (IBM Corp. IBM SPSS Statistics for Windows. Armonk, NY, USA), was used. The significance level was set to a two-sided probability (*p*) < 0.05.

## 3 Results

### 3.1 General

Baseline characteristics are presented in Table 1. Changes in medications between visits 1 and 2 occurred in 6 patients (beta blocker dose reduced in 4, digoxin dose reduced in 1, and angiotensinogen receptor blocker dose increased in 1). During AF, PWV could not be evaluated with the Sphygmocor in 2 patients, due to “not measurable” recordings indicated by the device (Table 2).

**Table 1:** Baseline characteristics.

Age, years	63 ± 8.0	
Male gender	29 ± 73	
BMI, kg/m^2^	29 ± 4.4	
Brachial SBP, mm Hg	123 ± 14.7	
Brachial DBP, mm Hg	75 ± 11.0	
Current smoker	3 ± 8	
Previous smoker	16 ± 40	
Comorbidity		
Hypertension	20 ± 50	
Diabetes mellitus type 2	6 ± 15	
CHD	1 ± 3	
Hyperlipidemia	9 ± 20	
Previous stroke	1 ± 3	
COPD	2 ± 5	
Treatments		
Previous DVT or PE	3 ± 8	
Treatment with beta blocker	37 ± 90	
Treatment with anticoagulants	35 ± 90	

Data presented as mean values ± SD or *n* (%), as appropriate. BMI, body mass index; SBP, systolic blood pressure; DBP, diastolic BP; CHD, coronary heart disease; COPD, chronic obstructive pulmonary disease; DVT deep venous thrombosis; PE, pulmonary embolism.

**Table 2:** Measurements in atrial fibrillation and sinus rhythm by the two-site methodology (SphygmoCor) and one site methodology (Arteriograph).

SphygmoCor	AF	SR	*p*	ICC	CI (ICC)
*n*	38				
PWV, m/s	9.3 ± 1.8	8.5 ± 1.6	<0.001	0.81	0.45–0.92
AIx, mm Hg	18.1 ± 9.1	24.9 ± 9.0	<0.001	0.56	0.05–0.79
cSBP, mm Hg	108.0 ± 13.1	103.3 ± 12.9	0.02	0.71	0.44–0.85
cDBP, mm Hg	75.3 ± 13.1	68.7 ± 9.3	0.01	0.33	−0.17–0.63
cMAP, mm Hg	89.0 ± 11.1	83.1 ± 10.2	<0.001	0.66	0.28–0.83
cPP, mm Hg	34.2 ± 6.1	34.8 ± 8.4	0.61	0.70	0.44–0.84
HR, bpm	79.7 ± 16.1	60.0 ± 9.8	<0.001	n/a	n/a
Arteriograph	AF	SR	*p*	ICC	CI (ICC)
*n*	40				
PWV, m/s	10.1 ± 1.5	10.0 ± 1.8	0.52	0.74	0.51–0.86
AIx, mm Hg	20.1 ± 13.3	29.2 ± 12.6	<0.001	0.61	0.12–0.81
cSBP, mm Hg	114.8 ± 16.7	110.6 ± 15.1	0.09	0.71	0.45–0.85
cDBP, mm Hg	73.1 ± 10.2	67.4 ± 10.0	<0.001	0.66	0.27–0.83
cMAP, mm Hg	87.0 ± 11.8	81.8 ± 10.9	0.01	0.67	0.35–0.83
cPP, mm Hg	41.7 ± 9.9	43.2 ± 10.4	0.27	0.76	0.55–0.88
HR, bpm	76.2 ± 17.0	60.7 ± 10.1	<0.001	n/a	n/a
brSBP, mm Hg	121.1 ± 12.9	114.9 ± 13.8	<0.001	0.72	0.42–0.86
brDBP, mm Hg	73.6 ± 10.2	69.1 ± 9.5	<0.001	0.64	0.30–0.81
brMAP, mm Hg	89.4 ± 10.5	80.2 ± 21.0	0.05	0.35	−0.13–0.64

Data are presented as mean values ± SD. Interclass coefficients (ICC) are presented as mean values with 95% confidence intervals (CI). AF, atrial fibrillation; SR, sinus rhythm; cfPWV, carotid to femoral pulse wave velocity; AIx, augmentation index; cSBP, central systolic blood pressure; cDBP, central diastolic BP; cMAP, central mean arterial pressure; cPP, central pulse pressure; HR, heart rate; brSB, brachial systolic blood pressure; brDBP, brachial diastolic blood pressure; brMAP, brachial mean arterial pressure; n/a, not applicable.

### 3.2 Assessments of central BP and AIx

Central BP measurements (Table 2) showed lower central systolic and diastolic BP values in SR than in AF for the two-site methodology (SphygmoCor; −4.8 ± 12.0/−6.6 ± 14.1 mm Hg) and for the one-site methodology (Arteriograph; −4.1 ± 15.0/−5.7 ± 9.5 mm Hg). As expected, this rendered a reduction in central MAP in SR, as compared to AF for both methods, while central pulse pressure (PP) remained unchanged, due to the equal reductions in both SBP and DBP (Table 2). Brachial BP values are also given in Table 2 for comparative reasons. AIx values were higher in SR, as compared to AF, for both the SphygmoCor (+6.8 ± 9.1 mm Hg) and the Arteriograph (+9.1 ± 12.4 mm Hg) (Table 2).

#### 3.2.1 Associations of central MAP and HR to AIx in AF and in SR

Linear regressions to assess the independent (controlled for age, body mass index, and concomitant disease) relation of central MAP and HR to AIx in AF and in SR were performed. An independent relation between HR (but not central MAP) and AIx was seen both in AF and in SR with the SphygmoCor (in AF: *β* = −0.62, in SR: *β* = −0.57, both *p* < 0.001). With the Arteriograph there were independent relations of both central MAP and HR to AIx in both rhythms (MAP in AF: *β* = 0.32, *p* = 0.03, HR in AF *β* = −0.26, *p* = 0.04; MAP in SR *β* = 0.28, *p* = 0.04, HR in SR *β* = −0.43 *p* = 0.003).

#### 3.2.2 Assessments of the impact of changes in central MAP and HR between visits for AIx

To control for the changes in central MAP and HR between visits 1 (AF) and 2 (SR), the difference in these values between visits was used as covariates in linear regressions using AIx in AF as independent, and AIx in SR as dependent variable. Relations between AIx in AF and in SR were strengthened by adjusting for the difference in central MAP and HR (SphygmoCor: β from 0.50 to 0.77; Arteriograph: β from 0.54 to 0.85). AIx in SR was also dependent on the difference in HR with the SphygmoCor (*β* = 0.61, *p* < 0.001), AIx in SR by the Arteriograph was dependent on the difference in both central MAP and HR (*β* = 0.41 and 0.51, both *p* < 0.001).

### 3.3 Assessments of PWV

**Figure 1:**
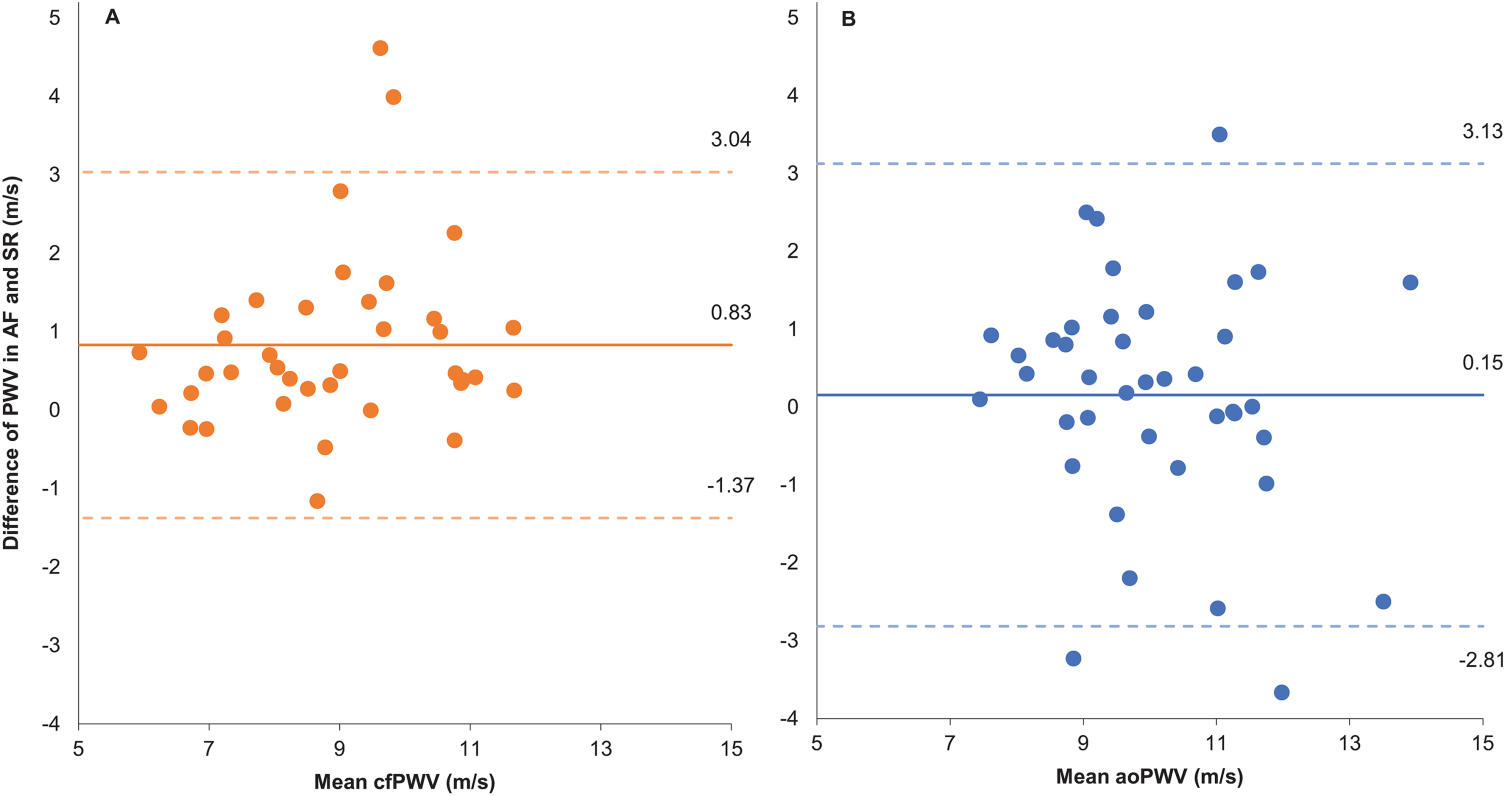
Bland-Altman plot. **(A)** Two-site methodology. **(B)** One-site methodology. Difference of pulse wave velocity (PWV) measured as AF – SR and mean PWV as the average PWV in AF and SR. AF, atrial fibrillation; SR, sinus rhythm; cfPWV, carotid to femoral PWV measured by two site method; aoPWV, aortic PWV estimated by one site method.

Measurements of cfPWV by the two-site methodology (SphygmoCor) were 0.83 ± 1.1 m/s higher during AF, as compared to SR (Table 2, Figure 1). Assessments of aoPWV using one-site methodology (Arteriograph) were similar during AF and SR (0.15 ± 1.5 m/s difference) (Table 2, Figure 1). ICC results were similar with both methodologies (Table 2).

#### 3.3.1 Associations of central MAP and HR to PWV in AF and in SR

Linear regressions to assess the independent (controlled for age, body mass index, and concomitant disease) relation of central MAP and HR to PWV in AF and in SR were performed. Central MAP and HR were related to cfPWV with the SphygmoCor in SR but not in AF (MAP in AF: *β* = 0.32, *p* = 0.09, HR in AF: *β* = 0.11, *p* = 0.50, MAP in SR: *β* = 0.48, *p* = 0.003; HR in SR: *β* = 0.30, *p* = 0.05). For assessed aoPWV with the Arteriograph these relations were slightly weaker to central MAP (MAP in AF: *β* = 0.25, *p* = 0.15; MAP in SR: *β* = 0.39, *p* = 0.01), and were not related to HR (HR in AF: *β* = 0.20, *p* = 0.20; HR in SR: *β* = 0.24, *p* = 0.10).

#### 3.3.2 Assessments of the impact of changes in central MAP and HR between visits for PWV

To control for the changes in central MAP and HR between visits 1 (AF) and 2 (SR), the difference in these values between visits was used as covariates in linear regressions using PWV in AF as independent, and PWV in SR as dependent variable. There were no changes in the strength of the relation between neither cfPWV nor aoPWV in AF and in SR when adjusting for changes in central MAP and HR with either device. However, the difference in central MAP was related to cfPWV in SR with the SphygmoCor (*β* = 0.27, *p* = 0.01).

### 3.4 Variability of measurements by the device algorithms and by number of repeated measurements

**Figure 2:**
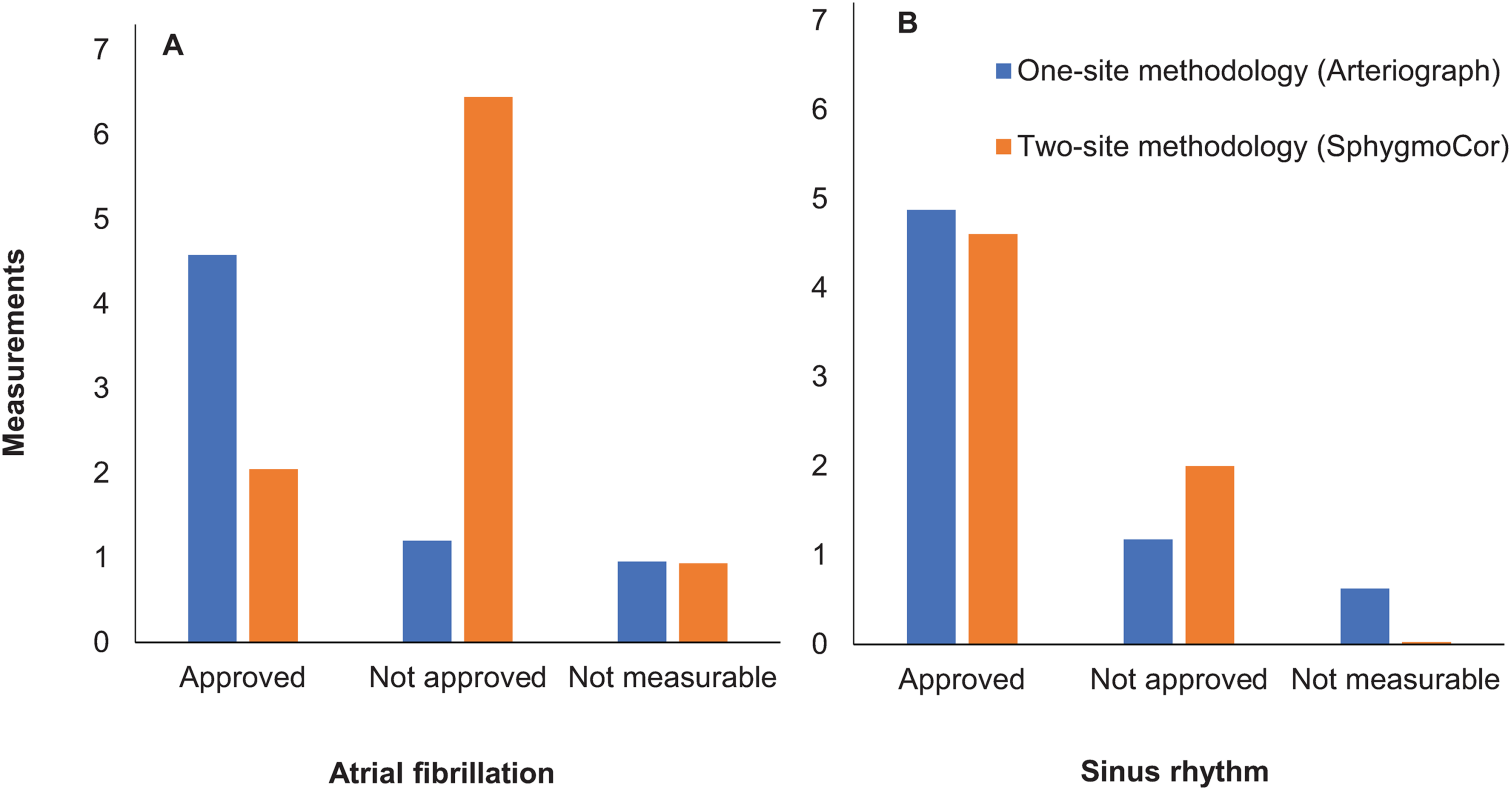
Number of performed measurements by the two methods in atrial fibrillation **(A)** vs. sinus rhythm **(B)**. Mean values of numbers of approved, not approved and not measurable recordings for pulse wave velocity are presented.

With the SphygmoCor, intraindividual variability (expressed and device calculated as Operator Index and SD; see Methods) was greater in AF than in SR, and was reflected in the high numbers of performed and low numbers of accepted measurements in AF (Table 3, Figure 2). The number of “not measurable” readings was low and similar in AF and SR.

**Table 3:** Numbers of performed measurements (mean), and device calculated standard deviations and operator Index.

SphygmoCor	AF	SR	*p*
*n*	38		
PWV measurements	9.5 ± 2.3	6.6 ± 1.7	<0.001
PWV approved	2.2 ± 2.4	4.6 ± 1.1	<0.001
PWV not approved	6.7 ± 3.2	2.0 ± 2.5	<0.001
PWV not measurable	0.7 ± 1.7	0.0 ± 0.2	0.01
PWV SD	2.5 ± 1.7	1.0 ± 0.5	<0.001
PWA measurements	9.8 ± 0.9	8.5 ± 2.5	0.004
PWA approved	1.3 ± 2.1	3.2 ± 2.0	<0.001
PWA not approved	8.4 ± 2.6	5.1 ± 3.4	<0.001
PWA not measurable	0.1 ± 0.4	0.2 ± 0.8	0.74
PWA OpIndex (%)	54.8 ± 16.5	71.0 ± 15.9	<0.001
Arteriograph	AF	SR	*p*
*n*	40		
Measurements	6.6 ± 1.9	6.7 ± 2.8	0.9
Approved	4.5 ± 1.1	4.9 ± 0.5	0.07
Not approved	1.0 ± 1.8	1.2 ± 2.1	0.69
Not measurable	1.1 ± 1.9	0.6 ± 2.0	0.29
SD	0.7 ± 0.2	0.6 ± 0.2	0.10

Data are presented as numbers of measurements ± SD. SD, standard deviation; PWV, pulse wave velocity; PWA, pulse wave analysis; OpIndex, Operator Index. Note that algorithms for calculating measurement variability as SD differ for the two devices and cannot be compared in absolute numbers, see Methods.

The intraindividual variability with the Arteriograph (expressed and device calculated as SD; see Methods), was similar in AF and SR, with lower numbers of performed and higher numbers of accepted measurements (Table 3, Figure 2). The numbers of “not measurable” readings in AF and SR were low.

**Figure 3:**
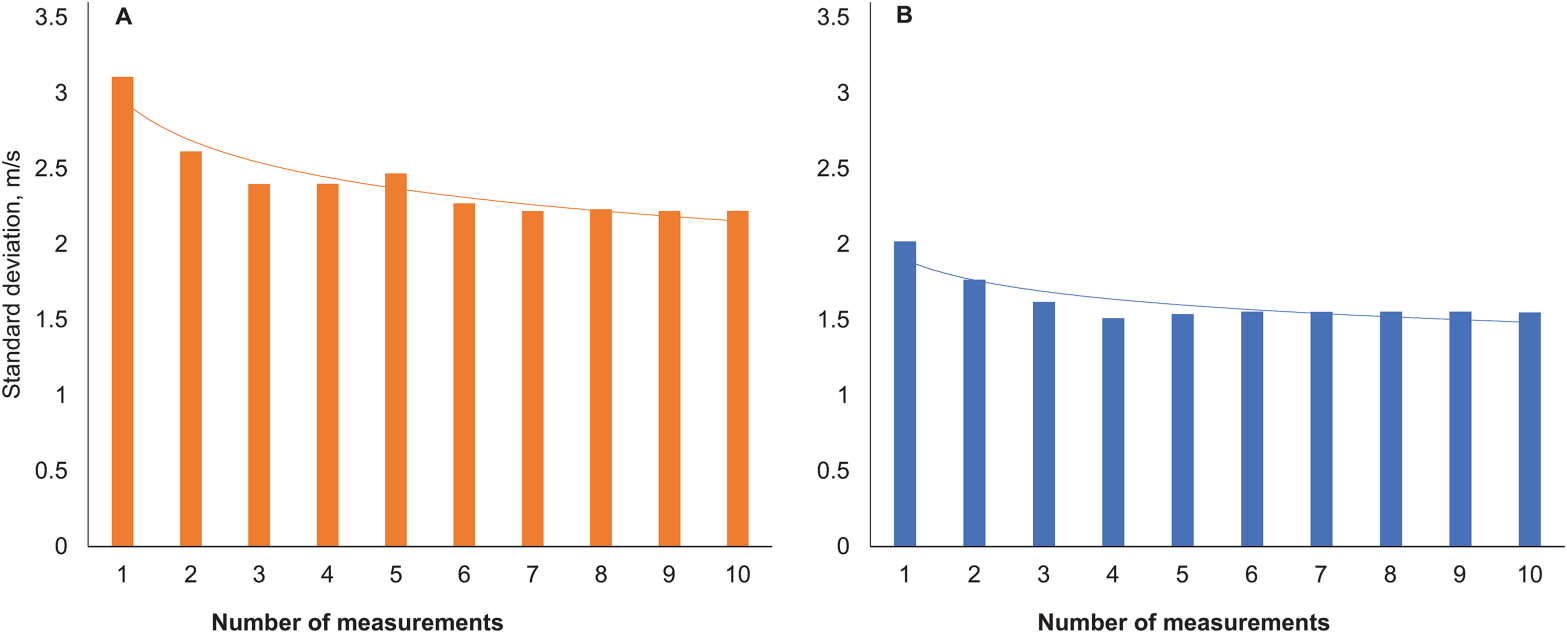
Standard deviations of pulse wave velocity with increasing numbers of measurements. Two-site method **(A)**, one-site method **(B)**.

The number of measurements to achieve a reliable estimate of cfPWV and aoPWV appeared to be 6–7 with two-site methodology and 4 with the one-site methodology (Figure 3).

## 4 Discussion

We studied whether aortic stiffness can be assessed reliably in patients during AF. A two-site ECG-triggered device using applanation tonometry (SphygmoCor) and a one-site methodology using oscillometric technique (Arteriograph) were employed in patients during AF and the results were compared intra-individually to recordings in SR after electric cardioversion. Our results show a decrease in central BP and HR, an increase in AIx, and similar values of PWV, suggesting that aortic stiffness can be non-invasively assessed during AF, provided a sufficient number of recordings are performed.

There are few studies on the reliability of measuring indices of aortic stiffness in AF. In the present study central systolic and diastolic BP decreased after electric cardioversion to SR. Results from intra-individual comparisons of brachial BP in AF and SR are not consistent but suggest a trend towards rhythm-dependent changes with lower systolic and higher diastolic BP in AF (28–31). If these changes are due to hemodynamic reasons or measurement errors remains unclear (32). However, our recent findings from intra-individual comparisons during ambulatory BP monitoring indicate that the beat-to-beat variations in BP in AF, as compared to SR, regress towards the mean with averaged values (25).

The calculation of AIx is dependent on central BP and, as expected, AIx increased after cardioversion from AF to SR in the present study. This is in agreement with an earlier report also using the SphygmoCor (33). Regression analyses showed a clear dependency on both BP and HR for AIx results, and relations between AIx in AF and SR were stronger in the adjusted models. Thus, an increase in AIx during SR, as compared to AF, should probably be interpreted as due to changes in BP and HR between visits. Of note, our findings on AIx with the two-site and the one-site methodology were consistent.

Caluwé et al. in a study like ours using the SphygmoCor device reported a BP dependent 1.1 m/s decrease in cfPWV after cardioversion from AF to SR (33). Those authors concluded that cfPWV measurements were reliable with the SphygmoCor, when adjusted for BP levels. Our study confirms and extends their findings by employing also a one-site single cuff-based oscillometric technique (Arteriograph) with beat-to-beat assessment of aortic stiffness measurements. Both methodologies provided similar results for PWV in AF and in SR with independent strength adjusted for the difference in BP and HR between AF and SR visits.

Our results with the two-site method on device-calculated intraindividual variability (both Operator Index for pulse waves and SD for measured PWV) (Figure 3) suggest that 6–7 repeated measurements in AF may be needed to reach stable cfPWV values. High indices of variability and low numbers of accepted measurements also in SR with this method was explained by many patients with frequent supraventricular extrasystoles during SR, which was almost as challenging for this procedure as persistent AF. Results with the one-site beat-to-beat method showed similar device-calculated intraindividual variability in AF and SR, an expected difference to the SphygmoCor device since the SphygmoCor algorithm is more sensitive to variability (see Methods). These results suggest that 4 repeated measurements in AF may be needed to assess indices of stiffness with the one-site beat-to-beat method.

There are some strengths with this study. We confirm that non-invasive assessment of aortic stiffness is feasible during AF and we provide new information on the use of a one-site beat-to-beat single cuff-based oscillometric method. Intraindividual comparisons in AF and SR with a short time frame between visits renders high statistical power and accounts for reducing potential confounding influence. There are also important limitations to consider. First, some individuals had greater visit to visit differences in the measured variables, which did not seem to be due to the magnitude of HR and BP changes. This study was not designed to determine precision on an individual level, which would have required a larger sample for further stratification on variables. However, the techniques investigated are primarily used in research, where comparisons on group level are more relevant. Second, uncertainties in BP measurement during AF affects the interpretation of our results. To our knowledge, no studies have yet reported invasive reference values for intraindividual comparisons of measures of BP in AF and SR. However, several non-invasive studies support the notion of regression towards the mean, making averaged BP values, especially MAP, and BP variability indices comparable in AF and SR (25, 34, 35). Third, the substantial difference in algorithms for calculating variability by the devices makes comparisons between methodologies difficult. Finally, there are no invasive studies of intraindividual comparison of PWV in AF and SR, and comparisons of invasive and non-invasive assessment of PWV during AF are missing, making assumptions for both comparisons in AF and SR and true variability of measurements in AF difficult. However, in the light of results indicating a regression towards the mean with BP in AF, it is plausible to believe that the same phenomenon might be true for aortic stiffness measures.

In conclusion, we show that it is feasible to measure aortic stiffness during AF with sufficient reliability to add useful information for cardiovascular risk assessment. The ECG-triggered two-site tonometry-based method (SphygmoCor) showed higher device calculated variability with lower numbers of approved readings necessitating several repeated measurements, than a one-site beat to beat oscillometric method (Arteriograph), using device calculated variability based on comparisons of several averaged values.

## Data Availability

The datasets presented in this article are not readily available as the dataset will only be shared for collaborative purposes to other academic researchers, and can for this purpose be used for further research questions/analyses, as long as these align with ethics approval for the study. Requests to access the datasets should be directed to kristina.lundwall@ki.se.
